# Exploration of ecological factors related to the spatial heterogeneity of tuberculosis prevalence in P. R. China

**DOI:** 10.3402/gha.v7.23620

**Published:** 2014-06-12

**Authors:** Xin-Xu Li, Li-Xia Wang, Juan Zhang, Yun-Xia Liu, Hui Zhang, Shi-Wen Jiang, Jia-Xu Chen, Xiao-Nong Zhou

**Affiliations:** 1 National Institute of Parasitic Diseases, Chinese Center for Disease Control and Prevention, Shanghai, People's Republic of China; 2 Key Laboratory of Parasite and Vector Biology, Ministry of Health, WHO Collaborating Centre for Malaria, Schistosomiasis and Filariasis, Shanghai, People's Republic of China; 3 National Center for Tuberculosis Control and Prevention, Chinese Center for Disease Control and Prevention, Beijing, People's Republic of China; 4 School of Mathematics and Physics, North China Electric Power University, Beijing, People's Republic of China; 5 Department of Epidemiology and Health Statistics, School of Public Health, Shandong University, Jinan, People's Republic of China

**Keywords:** tuberculosis, prevalence, ecological factor, spatial heterogeneity, P. R. China

## Abstract

**Background:**

The current prevalence of tuberculosis (TB) in the People's Republic of China (P. R. China) demonstrates geographical heterogeneities, which show that the TB prevalence in the remote areas of Western China is more serious than that in the coastal plain of Eastern China. Although a lot of ecological studies have been applied in the exploration on the regional difference of disease risks, there is still a paucity of ecological studies on TB prevalence in P. R. China.

**Objective:**

To understand the underlying factors contributing to the regional inequity of TB burden in P. R. China by using an ecological approach and, thus, aiming to provide a basis to eliminate the TB spatial heterogeneity in the near future.

**Design:**

Latent ecological variables were identified by using exploratory factor analysis from data obtained from four sources, i.e. the databases of the National TB Control Programme (2001–2010) in P. R. China, the China Health Statistical Yearbook during 2002–2011, the China Statistical Yearbook during 2002–2011, and the provincial government websites in 2013. Partial least squares path modelling was chosen to construct the structural equation model to evaluate the relationship between TB prevalence and ecological variables. Furthermore, a geographically weighted regression model was used to explore the local spatial heterogeneity in the relationships.

**Results:**

The latent ecological variables in terms of ‘TB prevalence’, ‘TB investment’, ‘TB service’, ‘health investment’, ‘health level’, ‘economic level’, ‘air quality’, ‘climatic factor’ and ‘geographic factor’ were identified. With the exception of TB service and health levels, other ecological factors had explicit and significant impacts on TB prevalence to varying degrees. Additionally, each ecological factor had different impacts on TB prevalence in different regions significantly.

**Conclusion:**

Ecological factors that were found predictive of TB prevalence in P. R. China are essential to take into account in the formulation of locally comprehensive strategies and interventions aiming to tailor the TB control and prevention programme into local settings in each ecozone.

As a major cause of illness and death, tuberculosis (TB) still spreads worldwide and is one of the most serious public health problems. The prevalence of TB is affected by various factors, including not only factors at the individual level but also factors at the ecological level. Regarding individual-level factors, those of genetic susceptibility (1), age (2), sex (3), race (4), socio-economic position (5, 6), occupation (7), smoking (8), drinking alcohol (9), related diseases such as diabetes mellitus (10, 11), HIV (12), silicosis (13), organ transplantation (14), and so on are associated with the prevalence of TB. At the ecological level, the nature and geographic factors such as sunshine exposure (15), elevation (16), climate (17), air pollution (18), and so on and the socio-economic factors such as ethnic differences (19), poverty (19, 20), national economical level (21, 22), national TB program budget allocation (23), and so on are found to have impacts on the TB prevalence. Compared with factors at the individual level (1–5, 7–10, 12–14) , there is still a paucity of ecological studies on TB prevalence worldwide, especially in the People's Republic of China (P. R. China) (15, 16, 18, 21–23). Therefore, it is essential to investigate the role of ecological factors on TB prevalence in order to provide important information for policy makers in the formulation of TB control and prevention strategies on a larger scale.

Over the past 20 years, P. R. China has successfully reduced the incidence rate of TB infections, halved the prevalence of the most infectious form of TB, and reduced TB mortality by 80%. The prevalence of sputum smear-positive TB declined from 134/100,000 population in 1990 to 47/100,000 population in 2010, and the TB mortality fell remarkably from 19/100,000 population in 1990 to 3.5/100,000 population in 2010 (24). However, the epidemiology of TB in P. R. China is geographically inequitable, which shows that the more remote areas in Western China have a more serious problem than the coastal plain in the East regarding TB prevalence. The rate of laboratory-confirmed (bacteriological positive pulmonary TB) TB in western provinces was more than three times the rate in eastern provinces and almost double that in central provinces (24). Thus, it is necessary to investigate the problems with respect to the geographical inequity of the TB prevalence and answer the question as to why this spatial heterogeneity of TB prevalence occurred in P. R. China.

In studying the regional difference of disease transmission risks, a simple cross-sectional ecological study is normally regarded as inferior to non-ecological designs, such as cohort and case-control studies. This is due to it being susceptible to the ecological fallacy (25). The confounding patterns of ecological fallacy are likely to be at play over time in the same geographical area and at the same time between different geographical areas. However, by combining secular and geographical variations, it is possible for the ecological study to weaken ecological fallacy to the most degree (25). In this regard, the present study was conducted to explore ecological causes for the spatial heterogeneity of TB prevalence by means of exploring the nature, geographic and socio-economic factors during 2001–2010 in different regions of P. R. China.

## Materials and methods

### Setting

In 2010, the Disease Control Bureau of the Ministry of Health and Chinese Center for Disease Control and Prevention implemented the 5th national TB epidemiological survey and evaluated the National Tuberculosis Control Programme (NTP) (2001–2010), which covered 31 provinces in P. R. China, and both reports were released in 2011 (24, 26). The data on TB prevalence in each province provided a useful perspective for the present study.

### Data sources

**Fig. 1:**
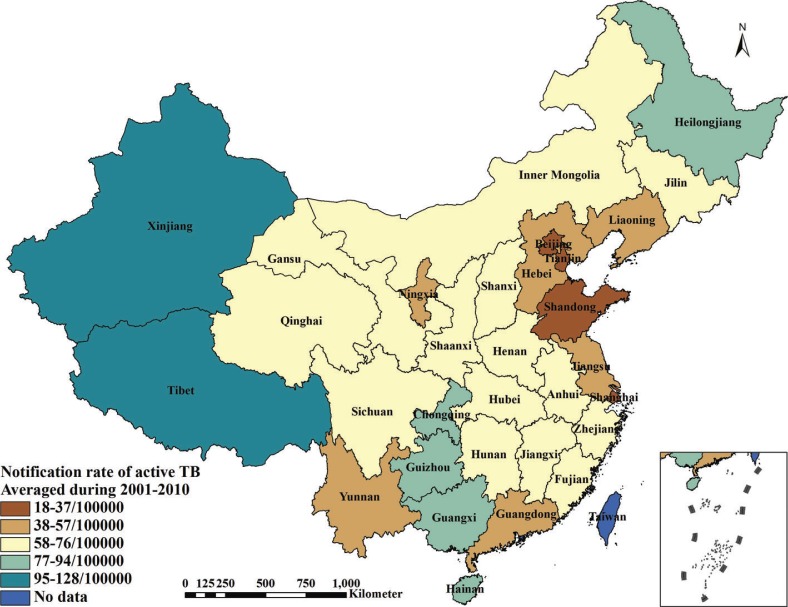
Averaged notification rate of active TB during 2001–2010 in P. R. China.

Data in terms of TB prevalence, investments for TB control and prevention and service level of TB control and prevention during 2001–2010 were extracted from the final evaluation report of NTP (2001–2010) in P. R. China (26). Data of investments for health work and health level of residents during 2001–2010 were collected from the P. R. China Health Statistical Yearbook during 2002–2011. The P. R. China Statistical Yearbook during 2002–2011 provided data of economic level, air quality and climatic factors during 2001–2010. Generally, the yearbook records information of the past year. Data of geographic factors were gathered from the provincial government websites in 2013. In order to increase the stability of data and minimize the potential bias, all of the collected data during 2001–2010 were averaged by province except the geographic factors, which was provided as a supplement (Supplementary file). Table 1 describes the observed variables and data sources. Figure 1 illustrates the average notification rate of active TB during 2001–2010.

**Table 1:** Specification of observed and latent variables

Observed variable	Description of observed variable	Data source	Period	Latent variable	% of variance^a^
NAT	Notification rate of active TB (1/100,000)	Final evaluation report of National Tuberculosis Programme (2001–2010) in China	2001–2010	TB prevalence	92.07
NNT	Notification rate of new sputum smear-positive TB (1/100,000)		2001–2010		
NST	Notification rate of sputum smear-positive pulmonary TB (1/100,000)		2001–2010		
LTP	Number of laboratory in TB control institutions per million people		2001, 2010	TB investment	72.79
PET	Per capita annual expenditure for TB control (RMB Yuan)		2001–2010		
STP	Number of staff in TB control institutions per 100,000 people		2001, 2005, 2010		
CNT	Cure rate of new sputum smear-positive TB cases (%)		2003–2009	TB service	65.79
CRT	Cure rate of relapse sputum smear-positive TB cases (%)		2003–2009		
TAR	Total arrival rate of the referral TB cases from non-TB control institutions (%)		2004–2010		

BMP	Number of bed in medical institutions per thousand people	China Health Statistical Yearbook	2001–2010	Health investment	83.68
MWP	Number of medical worker per thousand people		2001–2010		
PEH	Per capita annual expenditure for health work (RMB Yuan)		2001–2010		
LEP	Life expectancy (year)		2000, 2010	Health level	65.46
MMR	Maternal mortality rate (1/100,000)		2004–2010		
PDR	Population death rate (‰)		2001–2010		
PMR	Perinatal mortality rate (‰)		2003–2010		

GDP	Per capita gross domestic product (RMB Yuan)	China Statistical Yearbook	2001–2010	Economic level	96.25
PDI	Per capital annual disposable income of city households (RMB Yuan)		2001–2010		
PNI	Per capital annual net income of rural households (RMB Yuan)		2001–2010		
NO_2_	Annual concentration of nitrogen dioxide (mg/m^3^)		2003–2010	Air quality	61.91
PM10	Annual concentration of inhalable particulates (mg/m^3^)		2003–2010		
SO_2_	Annual concentration of sulphur dioxide (mg/m^3^)		2003–2010		
AAH	Annual average humidity (%)		2001–2010	Climatic factor	83.68
AAT	Annual average temperature (°C)		2001–2010		
APP	Annual precipitation (mm)		2001–2010		
AST	Annual sunshine time (hour)		2001–2010		

AEV	Average elevation (meter)	Provincial Government Websites	2013	Geographic	60.38
ALA	Average latitude (degree)		2013	factor	
ALO	Average longitude (degree)		2013		

a Exploratory factor analysis.

### Statistical methods

Exploratory factor analysis (EFA) was used to extract ecological factors that are latent and could not be measured directly from the aforementioned observed variables (27). The partial least squares path modelling (PLS-PM) was chosen to construct the structural equation modelling (SEM) to analyse the complex hypothesized causal relationship between TB prevalence and latent ecological factors (28, 29). This method was judged appropriate due to the consideration that the measured variables from 31 provinces in P. R. China presented characteristics of small sample size, non-normality, multidimension, and multicollinearity. Given that TB prevalence and latent ecological factors inevitably vary in different provinces, spatial examination of the causal relationships would play an important role in understanding the regional unbalance of TB prevalence. In this regard, the geographically weighted regression (GWR) model was employed to analyse the local spatial heterogeneity of the causal relationships between TB prevalence and latent ecological factors (30).

### Extraction of ecological variables

The latent ecological variables, including ‘TB prevalence’, ‘TB investment’, ‘TB service’, ‘health investment’, ‘health level’, ‘economic level’, ‘air quality’, ‘climatic factor’ and ‘geographic factor’, were extracted from the observed variables by exploiting the mean of EFA. Notification rate of active TB (NAT), notification rate of new sputum smear-positive TB (NNT) and notification rate of sputum smear-positive pulmonary TB (NST) were selected to denote the level of TB prevalence. Furthermore, TB investment was reflected by number of laboratory in TB control institutions per million people (LTP), per capita annual expenditure for TB control (PET) and number of staff in TB control institutions per 100,000 people (STP). TB service was represented by the cure rate of new sputum smear-positive TB cases (CNT), the cure rate of relapse sputum smear-positive TB cases (CRT) and the total arrival rate of the referral TB cases from non-TB control institutions (TAR). Health investment was denoted by the number of beds in medical institutions per thousand people (BMP), the number of medical workers per thousand people (MWP) and per capita annual expenditure for health work (PEH). Health level was evaluated by life expectancy (LEP), maternal mortality rate (MMR), population death rate (PDR) and perinatal mortality rate (PMR). Economic level was characterized by per capita gross domestic product (GDP), per capital annual disposable income of city households (PDI) and per capital annual net income of rural households (PNI). Air quality was reflected by annual concentration of nitrogen dioxide (NO_2_), annual concentration of inhalable particulates (PM10) and annual concentration of sulphur dioxide (SO_2_). Climatic factor was indicated by annual average humidity (AAH), annual average temperature (AAT), annual precipitation (APP) and annual sunshine time (AST); geographic factor was reflected by average elevation (AEV), average latitude (ALA) and average longitude (ALO). Table 1 presents all of the above-mentioned observed and latent variables in the present study in detail. Statistical Analysis System (SAS 9.2; SAS Institute Inc., Cary, NC, USA) was used for performing the EFA.

### Interplay analysis between TB prevalence and ecological variables

**Fig. 2:**
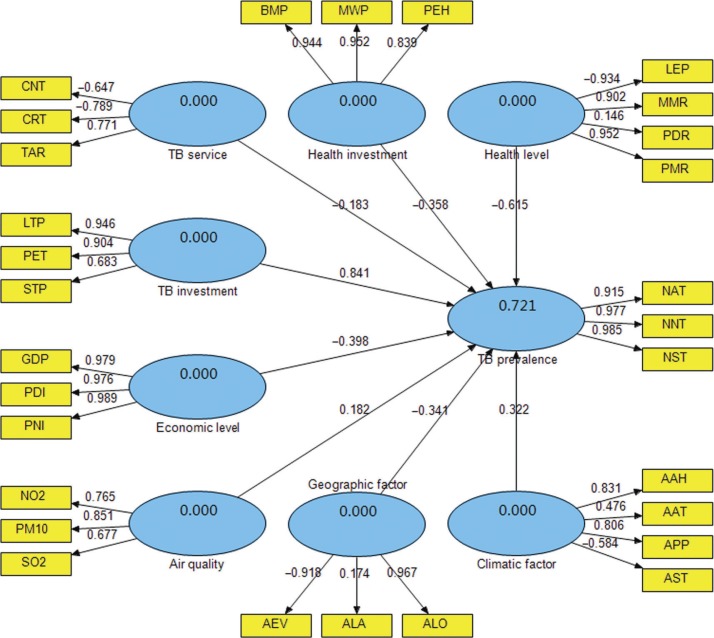
The partial least squares path model of TB prevalence with ecological factors.

Based on the results of the EFA, the PLS-PM was chosen to construct the SEM to evaluate the causal relationships between TB prevalence and ecological variables (Fig. 2). As a component-based estimation method, the PLS-PM works as an iterative algorithm that separately analyses the blocks of the measurement model and estimates the path coefficients in the structural model (28). In this study, SmartPLS (SmartPLS 2.0.M3; SmartPLS, Hamburg, Germany) was used for performing the PLS-PM, in which the path weighting scheme was implemented for the inner estimate of the standardized latent variable in the PLS procedure, and the default sample number for the bootstrapping procedure was 500. In the PLS-PM, the latent variable scores are estimated as exact linear combinations of their associated observed variables, and, therefore, treated as error free substitutes for the observed variables (28).

### Analysis for the local spatial heterogeneity of the causal relationships

Based on the latent variable scores in each province created by the PLS-PM, the GWR model was used to explore the local spatial heterogeneity in the causal relationships between TB prevalence and ecological factors. The GWR is an exploratory technique mainly intended to indicate where non-stationary is taking place on the map, namely exploring spatial heterogeneity (30). In this study, the local spatial regression model in terms of TB prevalence and ecological factors was set up by means of the GWR, whose regression coefficients express the local spatial variation and whose standard errors of coefficients indicate the reliability of the estimated coefficients. ArcGIS (ArcGIS 10.0; ESRI Inc., Redlands, CA, USA) was used for performing the GWR model and for creating the maps. The Natural Breaks (Jenks) method was used to classify the regression coefficients of ecological factors and their standard errors.

## Results

As demonstrated in Table 1 and Fig. 2, the latent variables reflected the related observed variables with varying degrees. The results showed that TB prevalence explained 92.1% of the total variance for NAT, NNT and NST. TB investment is followed, which explained 72.8% of the total variance for LTP, PET and STP. TB service explained 65.8% of the total variance for CNT, CRT and TAR. Nevertheless, CNT and CRT demonstrated the negative relationship with TB service. Health investment explained 83.7% of the total variance for BMP, MWP and PEH. Health level explained 65.5% of the total variance for LEP, MMR, PDR and PMR. However, LEP had the negative relationship with health level. Moreover, economic level explained 96.2% of the total variance for GDP, PDI and PNI. Air quality explained 61.9% of the total variance for NO_2_, PM10 and SO_2_. Climatic factor explained 83.7% of the total variance for AAH, AAT, APP and AST, while AST had the negative relationship with climatic factor. Geographic factor explained 60.4% of the total variance for AEV, ALA and ALO, but AEV had the negative relationship with geographic factor.

As shown in Fig. 2, eight latent variables explained 72.1% of the total variance for TB prevalence. Among them, TB investment had the most significant influence on TB prevalence with a standardized path coefficient 0.841, i.e. there was a positive relationship between TB investment and TB prevalence. Health level had the second largest effect on TB prevalence, with the coefficient −0.615, i.e. there was a negative relationship between health level and TB prevalence. Economic level, health investment, geographic factor and TB service all had negative effects on TB prevalence, with the coefficients −0.398, −0.358, −0.341 and −0.183, respectively. Climatic factor as well as air quality had positive effects on TB prevalence, with the coefficients 0.322 and 0.182, respectively.

Furthermore, Tables 2 and 3 show the bootstrapping test results for outer loading of the observed variables and path coefficient of the latent variables in the PLS-PM. The results demonstrate that all outer loadings and path coefficients were significant at a 0.05 level (all *P*<0.05).

**Table 2:** Bootstrapping test of outer loadings in the partial least square path model

Observed variable	Original sample (O)	Sample mean (M)	Standard deviation (STDEV)	Standard error (STERR)	T statistics (∣O/STERR∣)
NAT ← TB prevalence	0.9151	0.9153	0.0059	0.0059	155.9321***
NNT ← TB prevalence	0.9774	0.9771	0.0018	0.0018	543.2610***
NST ← TB prevalence	0.8312	0.7723	0.2473	0.2473	3.3607***
LTP ← TB investment	0.9017	0.9019	0.0045	0.0045	198.2337***
PET ← TB investment	0.9043	0.9037	0.0111	0.0111	81.7617***
STP ← TB investment	0.6832	0.6816	0.0275	0.0275	24.8022***
CNT ← TB service	−0.7885	−0.7687	0.0982	0.0982	8.0311***
CRT ← TB service	0.9788	0.9787	0.0018	0.0018	540.3380***
TAR ← TB service	0.7713	0.7752	0.0804	0.0804	9.5899***
BMP ← Health investment	−0.6474	−0.6268	0.1147	0.1147	5.6436***
MWP ← Health investment	0.7647	0.7527	0.0576	0.0576	13.2817***
PEH ← Health investment	0.8387	0.8349	0.0324	0.0324	25.8633***
LEP ← Health level	0.9458	0.9462	0.0047	0.0047	203.3511***
MMR ← Health level	0.9523	0.9528	0.0029	0.0029	328.4052***
PDR ← Health level	0.1463	0.1466	0.0482	0.0482	3.0360**
PMR ← Health level	0.9523	0.9515	0.0063	0.0063	150.7166***
GDP ← Economic level	−0.9336	−0.9334	0.0058	0.0058	159.6023***
PDI ← Economic level	0.9756	0.9754	0.0021	0.0021	468.4301***
PNI ← Economic level	0.9887	0.9887	0.0009	0.0009	1127.4221***
NO_2_ ← Air quality	0.9845	0.9843	0.0014	0.0014	685.4092***
PM10 ← Air quality	0.8509	0.8449	0.0478	0.0478	17.8010***
SO_2_ ← Air quality	0.6768	0.6472	0.1129	0.1129	5.9957***
AAH ← Climatic factor	0.4758	0.7006	0.2400	0.2400	1.9823*
AAT ← Climatic factor	−0.9183	−0.9170	0.0109	0.0109	84.6034***
APP ← Climatic factor	−0.5844	−0.7037	0.2238	0.2238	2.6116**
AST ← Climatic factor	0.9443	0.9441	0.0048	0.0048	195.4364***
AEV ← Geographic factor	0.1737	0.1729	0.0576	0.0576	3.0163**
ALA ← Geographic factor	0.9667	0.9662	0.0019	0.0019	496.5989***
ALO ← Geographic factor	0.8059	0.7385	0.2409	0.2409	3.3449***

* *P*<0.05

** *P*<0.01

*** *P*<0.001.

**Table 3:** Bootstrapping test of path coefficients in the partial least square path model

Latent variable	Original sample (O)	Sample mean (M)	Standard deviation (STDEV)	Standard error (STERR)	T Statistics (∣O/STERR∣)
TB investment → TB prevalence	0.8406	0.9035	0.1067	0.1067	7.8781***
TB service → TB prevalence	−0.1826	−0.2215	0.0614	0.0614	2.9728**
Health investment → TB prevalence	−0.3584	−0.3369	0.1372	0.1372	2.6122**
Health level → TB prevalence	−0.6146	−0.5350	0.1549	0.1549	3.9680***
Economic level → TB prevalence	−0.3982	−0.4045	0.1364	0.1364	2.9185**
Air quality → TB prevalence	0.1819	0.1952	0.0752	0.0752	2.4178*
Climatic factor → TB prevalence	0.3216	0.3413	0.1614	0.1614	1.9918*
Geographic factor → TB prevalence	−0.3410	−0.2155	0.1612	0.1612	2.1157*

* *P*<0.05

** *P*<0.01

*** *P*<0.001.

Table 4 summarizes the results of the GWR model between TB prevalence and the ecological factors, which indicated that there existed extensive spatial variations in parameters estimated from different province models. In the GWR model, R-squared equalled 0.776, which indicated that the model accounted for 77.6% of the variance for TB prevalence. Akaike information criterion with a correction (*AIC*c) for finite sample sizes, equalled 78.4. Moran's *I* for Residual equalled −0.040 and was of no significance at the 0.05 level (*P*=0.9264), which suggested that the residuals were spatially random.

**Table 4:** Parameter estimates of the geographical weighted regression model

Latent variable	Minimum	1st quartile	Median	3rd quartile	Maximum
Intercept	−0.0287	−0.0230	−0.0182	−0.0117	0.0103
TB investment	0.7213	0.7655	0.7954	0.8369	0.9285
TB service	−0.2908	−0.1513	−0.1270	−0.1109	−0.0982
Health investment	−0.3923	−0.3697	−0.3599	−0.3486	−0.3313
Health level	−0.7269	−0.6745	−0.6250	−0.5853	−0.4580
Economic level	−0.4879	−0.4540	−0.4306	−0.4113	−0.2937
Air quality	0.1144	0.1375	0.1568	0.1761	0.2591
Climatic factor	0.3102	0.3173	0.3177	0.3188	0.3228
Geographic factor	−0.3712	−0.3511	−0.3369	−0.3166	−0.1923

*R*^2^=0.776, adjusted *R*^2^=0.662, *AIC*c=78.433; Moran's *I* for Residual=−0.040, Z-score=−0.092, *P*=0.9264.

**Fig. 3:**
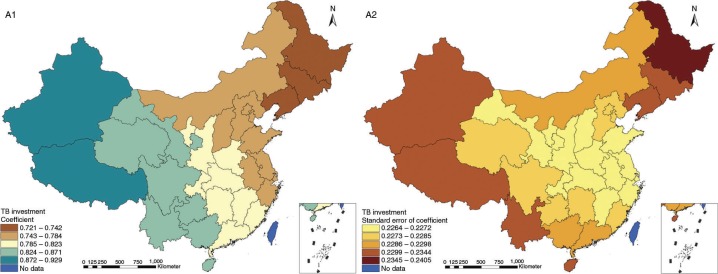
Spatial heterogeneity for coefficients of TB investment impacting on TB prevalence (A1: coefficient; A2: standard error of coefficient).

**Fig. 4:**
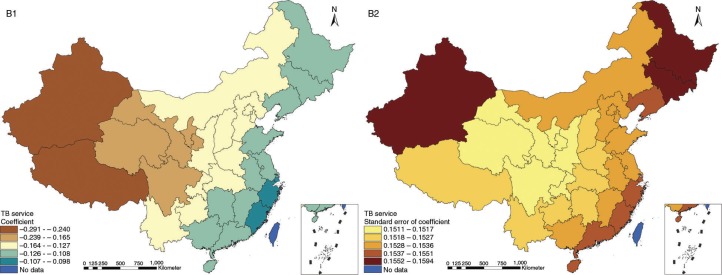
Spatial heterogeneity for coefficients of TB service impacting on TB prevalence (B1: coefficient; B2: standard error of coefficient).

**Fig. 5:**
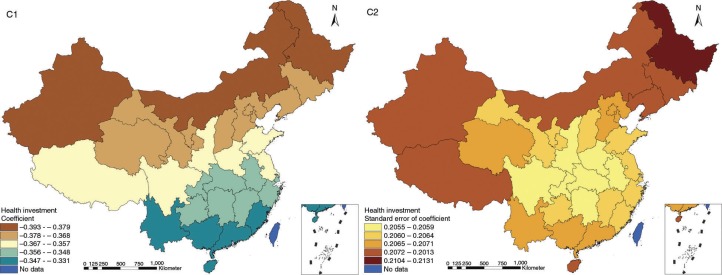
Spatial heterogeneity for coefficients of health investment impacting on TB prevalence (C1: coefficient; C2: standard error of coefficient).

**Fig. 6:**
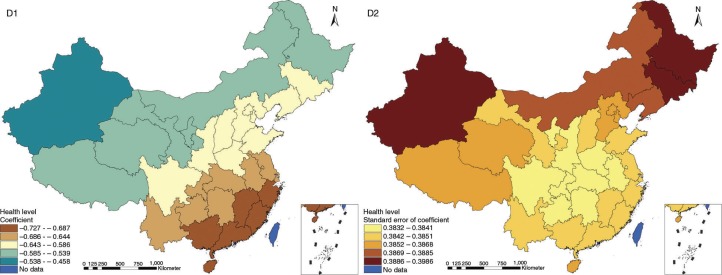
Spatial heterogeneity for coefficients of health level impacting on TB prevalence (D1: coefficient; D2: standard error of coefficient).

**Fig. 7:**
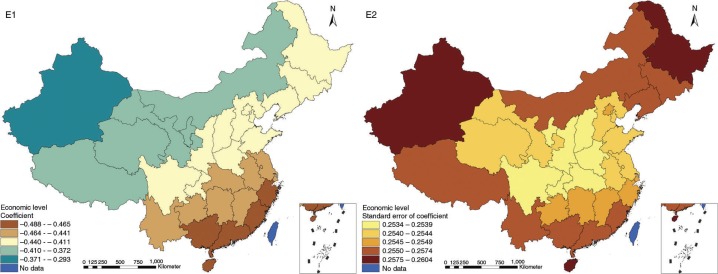
Spatial heterogeneity for coefficients of economic level impacting on TB prevalence (E1: coefficient; E2: standard error of coefficient).

**Fig. 8:**
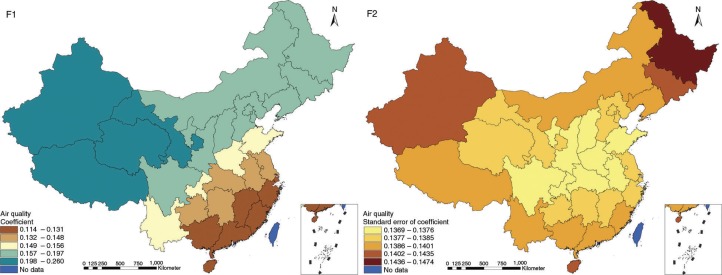
Spatial heterogeneity for coefficients of air quality impacting on TB prevalence (F1: coefficient; F2: standard error of coefficient).

**Fig. 9:**
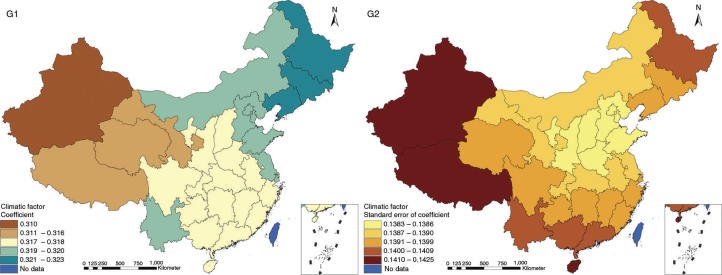
Spatial heterogeneity for coefficients of climatic factor impacting on TB prevalence (G1: coefficient; G2: standard error of coefficient).

**Fig. 10:**
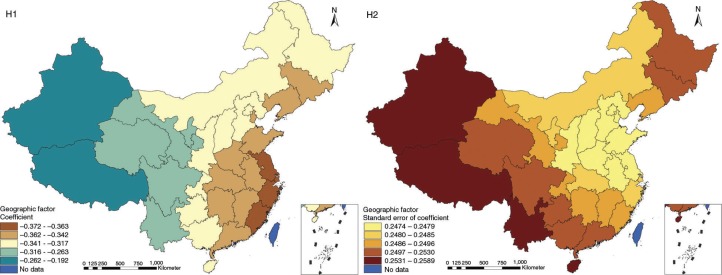
Spatial heterogeneity for coefficients of geographic factor impacting on TB prevalence (H1: coefficient; H2: standard error of coefficient).

The results in Figs. (3–10) show the contour map of the regression coefficients of ecological variables and their standard errors. TB investment had the largest positive effects on TB prevalence in Tibet and Xinjiang, with the standardized regression coefficients 0.872–0.929, and the lowest positive effects on TB prevalence in Liaoning, Jilin and Heilongjiang, with the coefficients 0.721–0.742 (Fig. 3). TB service had the largest negative effects on TB prevalence in Tibet and Xinjiang, with the coefficients −0.291 to −0.240, and the lowest negative effects on TB prevalence in Zhejiang and Fujian, with the coefficients −0.107 to −0.098 (Fig. 4). Health investment had the largest negative effects on TB prevalence in Inner Mongolia, Heilongjiang and Xinjiang, with the coefficients −0.393 to −0.379, and the lowest negative effects on TB prevalence in Fujian, Guangdong, Guangxi, Hainan and Yunnan, with the coefficients −0.347 to −0.331 (Fig. 5). Health level had the largest negative effects on TB prevalence in Zhejiang, Fujian, Jiangxi, Guangdong, Guangxi and Hainan, with the coefficients −0.727 to −0.687, and the lowest negative effects on TB prevalence in Xinjiang, with the coefficients −0.538 to −0.458 (Fig. 6). Economic level had the largest negative effects on TB prevalence in Zhejiang, Fujian, Guangdong, Guangxi and Hainan, with the coefficients −0.488 to −0.465, and the lowest negative effects on TB prevalence in Xinjiang, with the coefficients −0.371 to −0.293 (Fig. 7). Air quality had the largest positive effects on TB prevalence in Tibet, Gansu, Qinghai, and Xinjiang, with the coefficients 0.198–0.260, and the lowest positive effects on TB prevalence in Zhejiang, Fujian, Jiangxi, Guangdong, Guangxi and Hainan, with the coefficients 0.114–0.131 (Fig. 8). Climatic factor had the largest positive effects on TB prevalence in Liaoning, Jilin and Heilongjiang, with the coefficients 0.321–0.323, and the lowest positive effects on TB prevalence in Xinjiang, with the coefficients 0.310 (Fig. 9). Geographic factor had the largest negative effects on TB prevalence in Jiangsu, Zhejiang and Fujian, with the coefficients −0.372 to −0.363, and the lowest negative effects on TB prevalence in Tibet and Xinjiang, with the coefficients −0.262 to −0.192 (Fig. 10).

## Discussion

Although previous ecological studies adopted various methods to explore the relationships between ecological factors and TB prevalence, including Spearman's correlation coefficient analysis (20, 31), log-linear regression model (22), quintile regression model (21), multiple weighted linear regression model (19), general linear model (32), negative binomial regression model (33), mixed effects and generalized estimating equation models (23), and so on, these methods do not consider the internal relevance and potential structure of the factors (the construction of latent variables), which can be dealt with by the PLS-PM SEM (34). In this study, we used the PLS-PM SEM to estimate the relationships between ecological factors and TB prevalence. The PLS-PM is a soft-modelling-technique with minimum demands regarding measurement scales, sample sizes and residual distributions (28), and the SEM can fully use the data information and reveal the inner characteristics of observed factors comprehensively and thoroughly (29).

In our work, we found that TB investment, TB service, health investment, health level, economic level, air quality, climatic factor and geographic factor had impacts on TB prevalence with varying degrees. Due to the strategies of TB case detection, treatment and management to control and prevent TB in P. R. China (26), the more the province invested in TB control and prevention, the more TB cases were detected, which is consistent with the results of this study. Chapple et al. (23) also found that increasing the percentage of the NTP budget for advocacy, communication and social mobilization was associated with an increment in the TB case detection rate. In contrast, the more the investment for health work and the economic level, which represent the level of the province development, the less TB spread. These findings were similar with what the previous ecological studies have found (21, 22, 32, 33). Notably, we found that bad air quality, such as high concentrations of NO_2_, SO_2_ and inhalable particulates, can increase TB prevalence. Another study found that historical statistics supported a hypothesis linking TB and air pollution caused by coal, which was proposed whereby triggering of the interleukin-10 cascade by carbon monoxide in lung macrophages promotes the reactivation of *Mycobacterium tuberculosis* (18).

Although ecological factors related to the TB prevalence have not been well studied in P. R. China, we have found at least two important impact factors relevant to the TB prevalence. First, climatic factor had a complex impact on TB prevalence in the present study. It was estimated that the muggy weather (e.g. higher humidity, higher temperature, more precipitation and little sunshine exposure) can increase TB prevalence. Second, for a geographic factor, serious TB prevalence appeared in the areas with higher elevation, lower latitude and lower longitude in this study, which suggested that relatively serious TB prevalence occurred in western and southwestern China. These findings were similar with the results of the 5th national TB epidemiological survey in P. R. China, which showed that the areas with serious TB prevalence were mostly in Guangxi, Sichuan, Guizhou, Yunnan, Tibet and Xinjiang (35). Guangxi, Sichuan, Guizhou and Yunnan belong to the regions with the muggy weather. Therefore, besides the service level of TB control and prevention and the health level of residents, other ecological factors had explicit impacts on TB prevalence in P. R. China, which highlight the importance of comprehensive strategies and measures for TB control and prevention.

Although all eight ecological factors had demonstrated their impacts on TB prevalence in this study, their impacts were quite different. Specifically, the impact of investment for TB control and prevention was largest, and the second was the health level of residents. Furthermore, the results of the GWR model showed that each ecological factor had different impacts on TB prevalence in different regions. For the GWR model, higher values of R-squared (*R*^2^=0.776), lower *AIC*c values (*AIC*c=78.433) and spatially random distributions of the residuals (Moran's *I* for Residual=−0.040) demonstrated a better fit in this study (30). The contour map of the regression coefficients in the GWR model can be used to explain visually why there was regional unbalance of TB prevalence under the background of the uniform strategies and measures for TB control and prevention in P. R. China. The spatial variations of the ecological factors suggest that local strategies and measures for TB control and prevention should be formulated based on the spatial characteristics of the ecological factors.

Finally, it is necessary to point out that our study had certain limitations. First, our data were extracted from multiple sources, such as reports, yearbooks and websites, whose contents were collected by different organizations with different methods. Thus, analysing the relationships between data from different sources may place certain bias with respect to the achieved results. Second, the observed variables were constrained by data sources and we could only choose what were available in data sources. Hence, some observed variables may not fully reflect the latent ecological factors. For example, it was thought that a better service level of TB control and prevention and a better health level of residents had a relationship with higher TB prevalence, which was difficult to be explained in this study. There was a similar confusion that a better health service had a relationship with a higher prevalence of drug-resistant TB in the study exploring the ecological factors in association with drug-resistant TB worldwide (34). In view of these limitations, the findings of this study should be interpreted carefully when generalized to the larger or different regions and compared with results from other studies.

## Conclusion

We found that investment for TB control and prevention, service level of TB control and prevention, investment for health work, health level of residents, economic level, air quality, climatic factors and geography impacted on TB prevalence to various extents. Moreover, each ecological factor had different impacts on TB prevalence in different regions significantly in P. R. China. In view of this, locally comprehensive strategies and measures for TB control and prevention should be formulated according to the characteristics of the ecological factors.
